# Associations between sarcopenia with asthmatic prevalence, lung function and comorbidity

**DOI:** 10.1186/s12877-022-03394-9

**Published:** 2022-08-24

**Authors:** Zhigang Hu, Yufeng Tian, Xinyu Song, Fanjun Zeng, Ailan Yang

**Affiliations:** 1 Department of Respiratory and Critical Care Medicine, The First College of Clinical Medicine Science, China Three Gorges University, Yichang, 443003 People’s Republic of China; 2 Department of Respiratory and Critical Care Medicine, Yichang Central People’s Hospital at Zhijiang, NO. 183 Yiling Road, Zhijiang, 443003 People’s Republic of China; 3 Department of Respiratory and Critical Care Medicine, Yichang Central People’s Hospital, Yichang, 443003 People’s Republic of China; 4 Department of Academic Management, Clinical Research Center, China Three Gorges University, NO. 183 Yiling Road, Yichang, 443003 People’s Republic of China

**Keywords:** *Sarcopenia*, Asthma, Prevalence, Lung function, Comorbidity

## Abstract

**Background:**

Sarcopenia is listed as a treatment trait in behavioral/risk factors for severe asthma, but studies on asthma and sarcopenia are lacking. This study aimed to determine the associations between sarcopenia with asthmatic prevalence, symptoms, lung function and comorbidities.

**Methods:**

Fifteen thousand four hundred four individuals from the China Health and Retirement Longitudinal Study(CHARLS) and 10,263 individuals from the Study on global AGEing and adult health(SAGE) in China were included in this study. Four components of this study were used to assess the bidirectional association in the prevalence between sarcopenia with asthma, and estimate the relationships between sarcopenia with asthmatic symptoms, lung function and comorbidities via generalized additive models. The 10-item Center for Epidemiological Studies–Depression Scale ≥ 12 scores was classified as depression.

**Results:**

In the CHARLS and SAGE, the prevalence of sarcopenia in asthmatics was higher than those without asthma. Asthmatics with sarcopenia had a significantly increased prevalence of severe shortness of breath(sarcopenia yes vs. no, adjusted OR = 3.71, 95%CI: 1.43–9.60) and airway obstruction in the SAGE(sarcopenia yes vs. no, adjusted OR = 6.82, 95%CI: 2.54–18.34) and an obvious reduction of PEF in the CHARLS and SAGE(sarcopenia yes vs. no, adjusted RR = 0.86, 95%CI: 0.82–0.91) compared to asthmatics without sarcopenia. The presence of sarcopenia was positively associated with the prevalence of chronic obstructive pulmonary disease(sarcopenia yes vs no, adjusted OR = 5.76, 95%CI:2.01–16.5) and depression(sarcopenia yes vs no, adjusted OR = 1.87, 95%CI:1.11–3.14) in asthmatics.

**Conclusions:**

Our findings indicated that sarcopenia partakes in the development of asthma by affecting lung function and comorbidities and maybe considered a treatable trait of asthma management.

**Supplementary Information:**

The online version contains supplementary material available at 10.1186/s12877-022-03394-9.

## Introduction

In 2018, approximately 339 million individuals suffered from asthma with respiratory symptoms and sudden episodes of airflow limitation worldwide [1]. Frequent exacerbations of asthma place a substantial physiological and economic burden on patients and their families and can even lead to disability and mortality. Worsening lung function and comorbidities (such as depression and chronic lung diseases) are two independent risk factors for asthmatic exacerbations [1]. In recent years, treatable traits have been regarded as a new paradigm for asthma management, with one key paradigm involving the early identification of pulmonary, extrapulmonary, and behavioral/risk factors related to asthmatic exacerbations [2–5]. A recent study demonstrated that targeting treatable traits may reduce acute exacerbations, and improve the quality of life and asthma control in individuals with severe asthma [2].

Sarcopenia is defined as an age-related loss of skeletal muscle mass plus loss of muscle strength and/or reduced physical performance with the age cut-off values at either 60- or 65-years-old in the Asian Working Group for Sarcopenia (AWGS) 2019 [6]. The diagnosis of sarcopenia requires to have the measurement of appendicular skeletal muscle mass(ASM), muscle strength and physical performance. According to the AWGS 2014 criteria, the prevalence of sarcopenia ranges from 5.5% to 25.7% with a male predominance. The AWGS 2019 criteria recommended that the cut-off value of gait speed has increased from 0.8 m/s to < 1.0 m/s [6], which may increase the prevalence of sarcopenia. Sarcopenia with muscle fiber atrophy may cause immune senescence [7], the deterioration of respiratory force generation [8] and pulmonary function [9], frailty [10], depression and mortality [10] in the general population. In the study of McDonald et al., sarcopenia was regarded as a treatment trait in behavioral/risk factors for severe asthma. However, studies about the associations between sarcopenia with asthmatic prevalence, lung function and comorbidities are lacking.

It can be hypothesized that muscle fiber atrophy and weakness secondary to sarcopenia can potentially lead to the impairment of respiratory function, physical activity limitations and the increasing risk of developing depression or comorbidities in individuals with asthma. By using two national population-based studies in China, we evaluated the associations in the prevalence between sarcopenia with asthma. Subsequently, this study determined whether sarcopenia and its severity are associated with the reduction of lung function in elderly individuals with asthma. Finally, we investigated whether sarcopenia has significant relationships with comorbidities via generalized additive models.

## Methods

### Study population

The China Health and Retirement Longitudinal Study(CHARLS) was initiated between June 2011 and March 2012 and involved 17,708 individuals aged more than 45 years, which represented a national population-based health, social and economic status with encompassing 450 urban or rural areas in 28 provinces of China. The CHARLS used a face-to-face computer-assisted personal interview (CAPI) with physical measurements, blood sample collection, and depression assessments. Follow-ups were conducted every 2 years with the participation of new individuals in the CHARLS. The Biomedical Ethics Review Committee of Peking University approved the CHARLS. Written informed consent was collected at the National School of Development of Peking University. More detailed description of the CHARLS has been reported elsewhere [11] and can be found via the following link: https://charls.charlsdata.com/pages/Data/2018-charls-wave4/zh-cn.html (accessed on 30 May 2022).

The Study on global AGEing and adult health(SAGE) was designed by the World Health Organization(WHO), and this initiative planed to assess and compare the health status and socioeconomic consequences of adult populations and the ageing process in six countries(China, Ghana, India, Mexico, Russian Federation and South Africa) worldwide. The SAGE in China wave 1 was conducted between 2007 and 2010, involved 15,050 individuals and covered the eight provinces. In 2012, the data involving 19 districts in Shanghai and 9524 individuals were included in the SAGE. The WHO Ethical Review Committee and local ethics research review boards approved ethical and obtained written informed consent. ALL of the information and data can be found in a previous study [12] and via the following link: https://apps.who.int/healthinfo/systems/surveydata/index.php/catalog/sage (accessed on 30 May 2022).

### Definition of asthma, sarcopenia and depression

The diagnosis of asthma was based on a positive answer of the following question: “Have you ever been diagnosed with asthma by a doctor”. In the WHO SAGE, we collected the relevant data about asthmatic medication treatments and symptoms (attacks, awakening, and severe shortness of breath) in the past 12 months. Severe shortness of breath was based on a positive answer of the following question: “Have you had an attack of shortness of breath that came on without obvious cause when you were not exercising or doing some physical activity?”.

According to the recommendation of the AWGS 2019 [6], muscle strength and physical performance in the diagnosis of sarcopenia were measured by using handgrip strength (< 28.0 kg for men and < 18.0 kg for women) and gait speed (< 1.0 m/s) in the CHARLS and WHO SAGE. In addition, the CHARLS also provided 5-time chair stand test to measure low physical performance(≥ 12 s). The following anthropometric equation for the height-adjusted muscle mass (ASM/Ht^2^) can be used to determine whether low ASM existed in the Chinese population [6, 13]: ASM/Ht^2^ = (0.193*body weight + 0.107* height- 4.157* gender -0.037*age-2.631)/ height^2^. Similar to previous studies [14–16], the cut-off value for defining low muscle mass was based on the ASM/Ht2 of the lowest 20th percentile of the study population. Therefore, ASM/Ht^2^ < 6.92 for men and < 5.14 for female were regarded as low ASM. The age cut-off value of sarcopenia was set at 60-years-old [6]. Low ASM with low muscle strength or physical performance were defined as sarcopenia, meanwhile patients with severe sarcopenia were associated with low ASM, muscle strength and physical performance [6].

The CHARLS assessed whether depression existed by using the 10-item Center for Epidemiological Studies–Depression Scale (CES-D10). The 10 items with 4 four answer options were used to estimate the depressive feelings and behaviors of individuals over one week, and a value of 0–3 was assigned to each answer. Previous studies identified that the CES-D10 harbors adequate reliability and validity in the assessment of depression for the community-dwelling older Chinese population [17]. The CES-D10 ≥ 12 scores with total scores ranging from 0 to 30 scores were considered to indicate depression [17, 18].

### Variables

In the CHARLS, this study included the following demographic characteristics as adjusted confounding factors: sex, age, region, urban/rural, marital status, alcohol use, smoking, body mass index(BMI), night sleep duration and thirteen physician- diagnosed comorbidities (hypertension, dyslipidemia, hyperglycemia, cancers, chronic lung diseases, liver diseases, heart diseases, stroke, kidney diseases, digestive diseases, emotional or psychiatric problems, memory-related diseases, and arthritis or rheumatism). Lung function was measured through peak expiratory flow(PEF) in the CHARLS.

In the WHO SAGE, the following variables were used to adjust the associations between asthma and sarcopenia: sex, age, region, urban/rural, married status, alcohol, smoking, vigorous-intensity activity, moderate-intensity activity, BMI, night sleep duration, hypertension, diabetes, angina, stroke, chronic lung diseases and arthritis. This study evaluated lung function in individuals with asthma through the following variables: forced expiratory volume in the first second (FEV1), FEV1/forced vital capacity(FVC), PEF, forced expiratory flow rate, mid-exhalation (FEF25%–75%). FEV1/FVC < 0.7 was regarded as airway obstruction. Individuals with chronic lung diseases and FEV1/FVC < 0.7 were diagnosed as having chronic obstructive pulmonary disease (COPD).

For Chinese adults, BMI was divided into four groups: underweight (< 18.5 kg/m^2^), normal (18.5 to < 24.0 kg/m^2^), overweight (24.0 to < 28.0 kg/m^2^), and obesity (≥ 28.0 kg/m^2^) [19]. Age was divided into three groups: 60–69 years, 70–79 years, and 80 years and above. More detailed groups of all the variables are shown in Tables 1 and 2.

**Table 1:** The characteristics of study population in the China Health and Retirement Longitudinal Study

	No sarcopenia	Non-severe sarcopenia	Severe sarcopenia	*P*
N	12,159	2350	895	
Age	66.7 ± 5.6	69.9 ± 6.7	73.9 ± 7.2	< 0.01
Year				< 0.01
2011	3604 (29.6%)	877 (37.3%)	310 (34.6%)	
2013	3846 (31.6%)	715 (30.4%)	259 (28.9%)	
2015	4709 (38.7%)	758 (32.3%)	326 (36.4%)	
Sex				0.09
Male	6303 (51.8%)	1199 (51.0%)	495 (55.3%)	
Female	5856 (48.2%)	1151 (49.0%)	400 (44.7%)	
Region				< 0.01
Southwest	3271 (26.9%)	924 (39.3%)	322 (36.0%)	
South and central	6344 (52.2%)	1163 (49.5%)	447 (49.9%)	
North	2544 (20.9%)	263 (11.2%)	126 (14.1%)	
Urban/Rural				< 0.01
Urban	4927 (40.5%)	604 (25.7%)	224 (25.0%)	
Rural	7232 (59.5%)	1746 (74.3%)	671 (75.0%)	
Married status				< 0.01
Current unmarried	2021 (16.6%)	551 (23.4%)	302 (33.7%)	
Current married	10,138 (83.4%)	1799 (76.6%)	593 (66.3%)	
Alcohol				< 0.01
More than once a month	3170 (26.1%)	605 (25.7%)	220 (24.6%)	
Less than once a month	927 (7.6%)	144 (6.1%)	48 (5.4%)	
Never	8062 (66.3%)	1601 (68.1%)	627 (70.1%)	
Smoking				< 0.01
Never	6806 (56.0%)	1175 (50.0%)	456 (50.9%)	
Ever	1556 (12.8%)	239 (10.2%)	119 (13.3%)	
Current	3797 (31.2%)	936 (39.8%)	320 (35.8%)	
Body mass index category				< 0.01
Underweight	76 (0.6%)	834 (35.5%)	344 (38.4%)	
Normal	5996 (49.3%)	1512 (64.3%)	549 (61.3%)	
Overweight	4460 (36.7%)	4 (0.2%)	2 (0.2%)	
Obesity	1627 (13.4%)	0 (0.0%)	0 (0.0%)	
Night sleep duration				< 0.01
< 360 min	4000 (32.9%)	931 (39.6%)	370 (41.3%)	
360–419 min	2641 (21.7%)	459 (19.5%)	148 (16.5%)	
420–479 min	2150 (17.7%)	308 (13.1%)	107 (12.0%)	
480–539 min	2352 (19.3%)	427 (18.2%)	160 (17.9%)	
≥ 540 min	1016 (8.4%)	225 (9.6%)	110 (12.3%)	
Hypertension				< 0.01
No	8392 (69.0%)	1854 (78.9%)	675 (75.4%)	
Yes	3767 (31.0%)	496 (21.1%)	220 (24.6%)	
Dyslipidemia				< 0.01
No	10,637 (87.5%)	2185 (93.0%)	828 (92.5%)	
Yes	1522 (12.5%)	165 (7.0%)	67 (7.5%)	
Hyperglycemia				< 0.01
No	11,167 (91.8%)	2243 (95.4%)	858 (95.9%)	
Yes	992 (8.2%)	107 (4.6%)	37 (4.1%)	
Cancer				0.69
No	12,054 (99.1%)	2331 (99.2%)	885 (98.9%)	
Yes	105 (0.9%)	19 (0.8%)	10 (1.1%)	
Chronic lung diseases				< 0.01
No	10,846 (89.2%)	1999 (85.1%)	773 (86.4%)	
Yes	1313 (10.8%)	351 (14.9%)	122 (13.6%)	
Liver diseases				0.002
No	11,608 (95.5%)	2271 (96.6%)	871 (97.3%)	
Yes	551 (4.5%)	79 (3.4%)	24 (2.7%)	
Heart diseases				< 0.01
No	10,316 (84.8%)	2069 (88.0%)	786 (87.8%)	
Yes	1843 (15.2%)	281 (12.0%)	109 (12.2%)	
Stroke				0.02
No	11,821 (97.2%)	2308 (98.2%)	867 (96.9%)	
Yes	338 (2.8%)	42 (1.8%)	28 (3.1%)	
Kidney diseases				0.48
No	11,329 (93.2%)	2199 (93.6%)	842 (94.1%)	
Yes	830 (6.8%)	151 (6.4%)	53 (5.9%)	
Digestive diseases				0.01
No	9527 (78.4%)	1785 (76.0%)	680 (76.0%)	
Yes	2632 (21.6%)	565 (24.0%)	215 (24.0%)	
Emotional, nervous, or psychiatric problems				0.10
No	11,997 (98.7%)	2327 (99.0%)	889 (99.3%)	
Yes	162 (1.3%)	23 (1.0%)	6 (0.7%)	
Memory-related diseases				0.76
No	11,907 (97.9%)	2306 (98.1%)	875 (97.8%)	
Yes	252 (2.1%)	44 (1.9%)	20 (2.2%)	
Arthritis or rheumatism				0.34
No	8044 (66.2%)	1584 (67.4%)	607 (67.8%)	
Yes	4115 (33.8%)	766 (32.6%)	288 (32.2%)	
Asthma				< 0.01
No	11,671 (96.0%)	2228 (94.8%)	846 (94.5%)	
Yes	488 (4.0%)	122 (5.2%)	49 (5.5%)	
Depression				< 0.01
No	8847 (72.8%)	1551 (66.0%)	588 (65.7%)	
Yes	3312 (27.2%)	799 (34.0%)	307 (34.3%)	
PEF	289.5 ± 119.5	237.5 ± 108.7	195.9 ± 105.3	< 0.01

**Table 2:** The characteristics of study population in the Study on global AGEing and adult health from China

	No sarcopenia	Non-severe sarcopenia	Severe sarcopenia	*P*
N	8998	753	512	
Age	68.8 ± 6.9	73.0 ± 7.2	76.1 ± 7.3	< 0.01
Sex				< 0.01
Male	4506 (50.1%)	312 (41.4%)	199 (38.9%)	
Female	4492 (49.9%)	441 (58.6%)	313 (61.1%)	
Region				0.01
North	2754 (30.6%)	195 (25.9%)	141 (27.5%)	
South	6244 (69.4%)	558 (74.1%)	371 (72.5%)	
Urban/Rural				< 0.01
Urban	4985 (55.4%)	297 (39.4%)	151 (29.5%)	
Rural	4013 (44.6%)	456 (60.6%)	361 (70.5%)	
Married status				< 0.01
Current unmarried	1790 (19.9%)	253 (33.6%)	205 (40.0%)	
Current married	7208 (80.1%)	500 (66.4%)	307 (60.0%)	
Alochol				0.05
Ever	2349 (26.1%)	175 (23.2%)	115 (22.5%)	
Never	6649 (73.9%)	578 (76.8%)	397 (77.5%)	
Smoking				0.73
Never	6302 (70.0%)	522 (69.3%)	371 (72.5%)	
Ever	888 (9.9%)	72 (9.6%)	44 (8.6%)	
Current	1808 (20.1%)	159 (21.1%)	97 (18.9%)	
Body mass index				< 0.01
Underweight	131 (1.5%)	231 (30.7%)	146 (28.5%)	
Normal	3992 (44.4%)	521 (69.2%)	364 (71.1%)	
Overweight	3525 (39.2%)	1 (0.1%)	2 (0.4%)	
Obesity	1350 (15.0%)	0 (0.0%)	0 (0.0%)	
Moderate-intensity activity				< 0.01
Yes	2840 (31.6%)	304 (40.4%)	188 (36.7%)	
No	6158 (68.4%)	449 (59.6%)	324 (63.3%)	
Vigorous-intensity activity				0.61
Yes	748 (8.3%)	66 (8.8%)	37 (7.2%)	
No	8250 (91.7%)	687 (91.2%)	475 (92.8%)	
Night sleep duration				< 0.01
< 360 min	751 (8.3%)	79 (10.5%)	62 (12.1%)	
360–419 min	1210 (13.4%)	92 (12.2%)	53 (10.4%)	
420-479 min	1958 (21.8%)	126 (16.7%)	58 (11.3%)	
480-539 min	2890 (32.1%)	224 (29.7%)	122 (23.8%)	
≥ 540 min	2189 (24.3%)	232 (30.8%)	217 (42.4%)	
Hypertension				< 0.01
No	5359 (59.6%)	586 (77.8%)	403 (78.7%)	
Yes	3639 (40.4%)	167 (22.2%)	109 (21.3%)	
Diabetes				< 0.01
No	8082 (89.8%)	718 (95.4%)	489 (95.5%)	
Yes	916 (10.2%)	35 (4.6%)	23 (4.5%)	
Stroke				0.22
No	8051 (89.5%)	685 (91.0%)	467 (91.2%)	
Yes	947 (10.5%)	68 (9.0%)	45 (8.8%)	
Angina				0.32
No	8486 (94.3%)	720 (95.6%)	483 (94.3%)	
Yes	512 (5.7%)	33 (4.4%)	29 (5.7%)	
Chronic lung diseases				< 0.01
No	8101 (90.0%)	654 (86.9%)	434 (84.8%)	
Yes	897 (10.0%)	99 (13.1%)	78 (15.2%)	
Arthritis				0.01
No	6879 (76.5%)	615 (81.7%)	376 (73.4%)	
Yes	2119 (23.5%)	138 (18.3%)	136 (26.6%)	
Asthma				0.1
No	8733 (97.1%)	724 (96.1%)	490 (95.7%)	
Yes	265 (2.9%)	29 (3.9%)	22 (4.3%)	
FEV1	1.7 ± 0.7	1.5 ± 0.7	1.3 ± 0.7	< 0.01
FEV1/FVC				< 0.01
≥ 0.7	7252 (80.6%)	555 (73.7%)	352 (68.8%)	
< 0.7	1746 (19.4%)	198 (26.3%)	160 (31.2%)	
PEF	78.6 ± 19.9	76.6 ± 19.5	73.8 ± 21.0	< 0.01
FEF25-75	2.0 ± 1.1	1.6 ± 1.1	1.3 ± 1.0	< 0.01

### Statistical analysis

Study populations were categorized into three groups: no sarcopenia, non-severe sarcopenia and severe sarcopenia. SPSS described the categorical variables by counts and percentages (%), subsequently compared the difference among the three groups via a chi-square test. Continuous variables are presented as means and standard deviations with the Mann–Whitney U test for skewed continuous variables and Student’s t test or one-way ANOVA for normally distributed continuous variables. The chi-square goodness-of-fit method was used to test the normality of the distribution of the data. The first component of the study assessed the associations in the prevalence between sarcopenia and asthma via three generalized additive models with binomial regression.The adjusted variables of each model were shown in each table. Model 1 included patient demographic characteristics, Model 2 added physical/behavioral factors, and Model 3 added physical/behavioral factors and comorbidities. In the CHARLS, the least absolute shrinkage and selection operator (LASSO) [20] and multivariate logistic analyses with binomial regression were used to screen the independent risk factors for sarcopenia in asthmatics. We also evaluated the relationships between sarcopenia with asthmatic symptoms in the second component. Model 4 also included lung function on the basis of model 3 in Table 3. The third component was that three generalized additive models with Poisson regression were used to compare the differences in lung function among the three sarcopenia groups in asthmatics. In the WHO SAGE, model 4 added the adjustments of asthmatic medications and symptoms. Finally, three generalized additive models with binomial regression were used to evaluate the associations between sarcopenia with depression and COPD in asthmatics. All statistical analyses were done in SPSS, Empower(R) (www. empowerstats.com; X&Y solutions, Inc., Boston MA). Odds ratios (ORs) for binomial regression analysis and rate ratios (RRs) for Poisson regression analysis with 95% confidence intervals (CIs) represented the strength of associations, meanwhile a two tailed *P* < 0.05 was considered statistically significant.

**Table 3:** The associations between sarcopenia and asthma-related symptoms in the Study on global AGEing and adult health from China

	Model 1	Model 2	Model 3	Model 4
Asthma-related attacks				
Sarcopenia(no vs yes)	0.97(0.40, 2.38)	0.82(0.32, 2.06)	0.72(0.28, 1.86)	0.53(0.19, 1.51)
Asthma-related awakening				
Sarcopenia(no vs yes)	1.72(0.69, 4.31)	1.97(0.74, 5.26)	2.02(0.73, 5.59)	2.66(0.90, 7.82)
Asthma-related severe shortness of breath				
Sarcopenia(no vs yes)	3.51(1.50, 8.20)^#^	3.63(1.50, 8.79)^#^	3.41(1.38, 8.42)^#^	3.71(1.43, 9.60)^#^

Model 1 adjusted the following variables: sex, age, region, urban/rural, married status and body mass index

Model 2 adjusted the following variables: sex, age, region, urban/rural, married status, body mass index, alcohol, smoking, vigorous-intensity activity, moderate- intensity activity and night sleep duration

Model 3 adjusted the following variables: sex, age, region, urban/rural, married status,body mass index, alcohol, smoking, vigorous-intensity activity, moderate- intensity activity, night sleep duration, hypertension, diabetes, angina, stroke, chronic lung diseases, and arthritis

Model 4 adjusted the following variables: sex, age, region, urban/rural, married status, alcohol, smoking, vigorous-intensity activity, moderate-intensity activity, body mass index, night sleep duration, hypertension, diabetes, angina, stroke, chronic lung diseases, arthritis, FEV1, airway obstruction, PEF, and FEF 25%-75%)

^#^ *P* < 0.01

## Results

### Characteristics of the CHARLS and WHO SAGE

In three cycles of the CHARLS (2011, 2013 and 2015), a total of 15,404 individuals aged 67.6 ± 6.2 years (range, 60–103 years) were included in this present study. More than half (51.9%) of individuals were male with predominantly rural (62.4%), never alcohol and smoking. The overall prevalence of sarcopenia was 21.1% with 5.8% representing severe sarcopenia. The prevalence of sarcopenia showed an upwards trend with increasing age: 14.4%, 31.8%, and 51.2% in the 60–69, 70–79 and ≥ 80 years groups, respectively. A total of 659 (4.3%) individuals reported physician- diagnosed asthma. In addition, a total of 4433 (28.7%) individuals were classified as depressive group (CES-D10 ≥ 12 scores).

A total of 10,263 individuals aged 69.5 ± 7.2 years(range, 60–102 years) were selected from the WHO SAGE. About 47% of individuals came from rural. Predominantly female (51.1%) with high proportions of never drinking alcohol and never smoking were shown in the WHO SAGE. The overall prevalence of sarcopenia was 12.3% with 10.2% among male and 14.4% among female. A total of 512(5%) individuals were diagnosed with severe sarcopenia. Moreover, the prevalence of sarcopenia in the three age groups also significantly increased and varied by 6.0%, 16.4% and 32.5%. A total of 2104 (20.5%) individuals had airway obstruction (FEV1/FVC < 0.7). Additionally, the prevalence of asthma was 3.1% (316 individuals). A total of 301 asthmatics provided the information about medication treatment (73.4%) and asthma-related symptoms. Furthermore, the proportions of asthma-related attacks, awakening, and severe shortness of breath were 68.1%, 27.2% and 42.2%, respectively. More detailed data were shown in Tables 1 and 2.

### Associations in the prevalence between sarcopenia with asthma

**Fig. 1:**
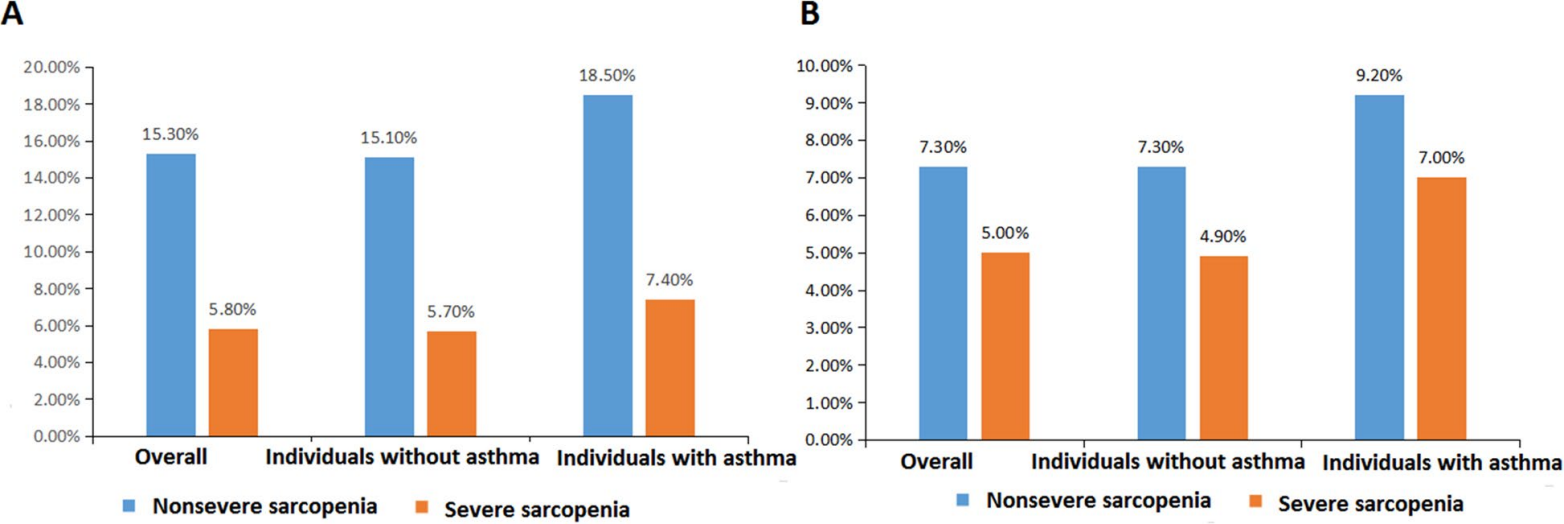
The prevalence of sarcopenia. **A** In the China Health and Retirement Longitudinal Study; **B** In the Study on global AGEing and adult health from China

In the CHARLS, the prevalence of non-severe and severe sarcopenia among asthmatics were 18.5% and 7.4%, respectively (see Fig. 1). A total of 4.0% of individuals with sarcopenia had a diagnosis of asthma, which was higher than that of individuals without sarcopenia (2.9%, *P* = 0.036). Model 3 indicated that sarcopenia and asthma had no significant bidirectional association in the prevalence (see Table S1). In asthmatics, the LASSO (see S1 Figure) and multivariate logistic regression analyses indicated that aged with rural (rural vs. unban, adjusted OR = 0.41, 95%CI: 0.23–0.75) and depression (CES-D10 < 12 scores vs. ≥ 12 scores, adjusted OR = 1.95, 95%CI: 1.16–3.27) harbored relatively high prevalence of sarcopenia.

In the WHO SAGE, 9.2% and 7% of individuals with asthma could be diagnosed with non-severe and severe sarcopenia, which were higher than those in individuals without asthma (see Fig. 1). The prevalence of asthma was 4.0% in individuals with sarcopenia. Three models observed no significant association in the prevalence between asthma and sarcopenia (see Table S2). The LASSO (see S1 Figure) and multivariate logistic regression analyses suggested that female, rural, aged and airway obstruction were independent risk factors for sarcopenia among asthmatics.

### Sarcopenia and asthma-related symptoms

**Fig. 2:**
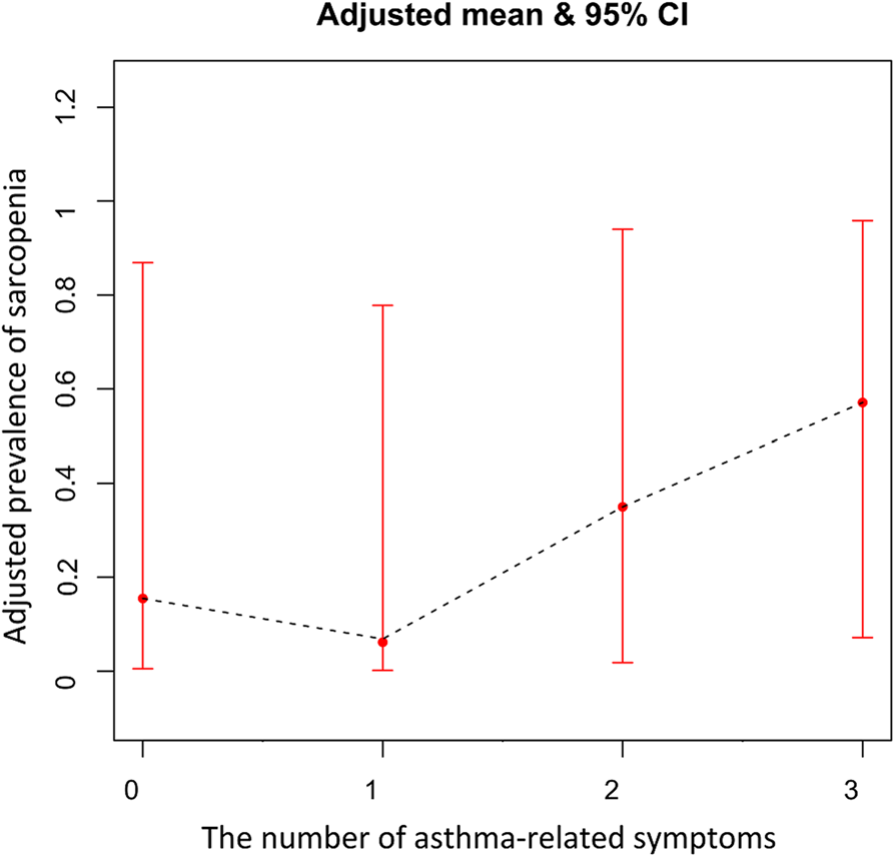
The associations between the prevalence of sarcopenia with the number of asthma-related symptoms in asthmatics from the Study on global AGEing and adult health from China

Four models also showed that asthma-related attacks and awakening had no significant associations with the presence of sarcopenia (see Table 3). However, sarcopenia was associated with a significantly higher prevalence of asthma-related severe shortness of breath after adjusting for demographic characteristics, physical/behavioral factors, comorbidities and lung function (adjusted OR = 3.71, 95%CI: 1.43–9.60). The prevalence of sarcopenia demonstrated a rising tendency with the increasing numbers of asthma-related symptoms (see Fig. 2).

### Sarcopenia and lung function in asthmatics

In the CHARLS, Poisson regression analyses suggested that the adjusted mean value of PEF was significantly declined in asthmatics with non-severe (adjusted RR = 0.926, 95%CI: 0.910–0.934) and severe sarcopenia (adjusted RR = 0.759, 95%CI: 0.739–779) compared to no sarcopenia (see Table S3).

**Fig. 3:**
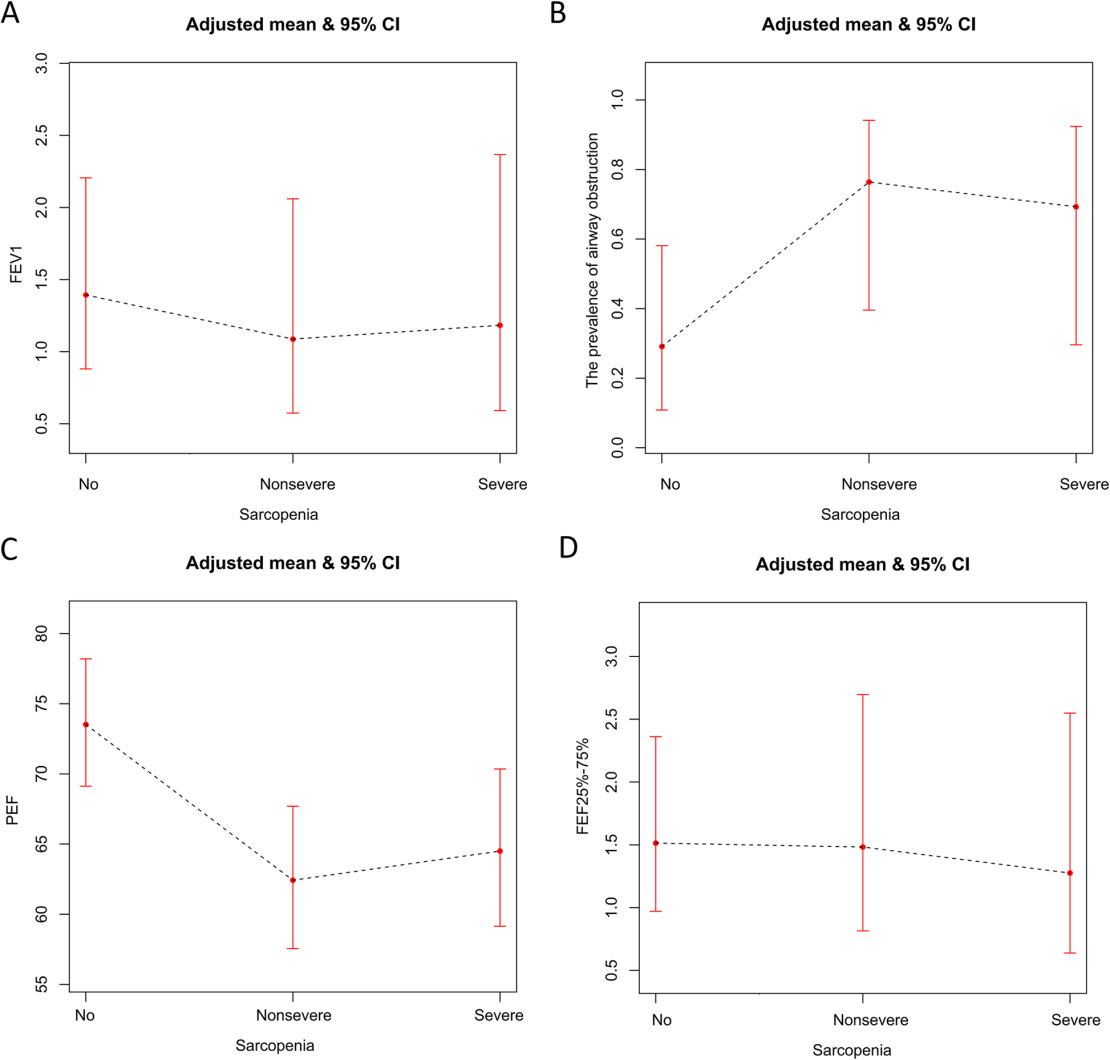
The associations between different grade of sarcopenia with lung function in asthmatics from the Study on global AGEing and adult health from China. **A** FEV1; **B** FEV1/FVC < 0.7; **C** PEF; **D** FEF 25%-75%

In the WHO SAGE, four Poisson regression analyses demonstrated that the more severe sarcopenia in asthmatics, the more obvious was lung function impairment (see Table 4 and Fig. 3). Asthmatics with sarcopenia (adjusted RR = 6.82, 95%CI: 2.54–18.34 in model 4) were associated with a significantly higher prevalence of airway obstruction than those without sarcopenia. Sarcopenia had a significantly negative correlation with the adjusted mean value of PEF in asthmatics (adjusted RR = 0.86, 95%CI: 0.82–0.91 in model 4).

**Table 4:** The associations between sarcopenia and lung function of asthmatics in the Study on global AGEing and adult health from China

	Model 1	Model 2	Model 3	Model 4
FEV1				
Sarcopenia(no vs yes)	0.75(0.57, 1.01)	0.75(0.50, 1.12)	0.80(0.53, 1.20)	0.81(0.53, 1.22)
FEV1/FVC < 0.7				
Sarcopenia(no vs yes)	5.44(2.25, 13.19)^#^	5.89(2.34, 14.82)^#^	5.37(2.09, 13.80)^#^	6.82(2.54, 18.34)^#^
PEF				
Sarcopenia(no vs yes)	0.87(0.82, 0.91)^#^	0.87(0.83, 0.92)^#^	0.89(0.85, 0.94)^#^	0.86(0.82, 0.91)^#^
FEF25%-75%				
Sarcopenia(no vs yes)	0.89(0.61, 1.30)	0.88(0.60, 1.29)	0.96(0.66, 1.42)	0.93(0.63, 1.38)

Model 1 adjusted the following variables: sex, age, region, urban/rural, married status and body mass index

Model 2 adjusted the following variables: sex, age, region, urban/rural, married status, body mass index, alcohol, smoking, vigorous-intensity activity, moderate- intensity activity and night sleep duration

Model 3 adjusted the following variables: sex, age, region, urban/rural, married status,body mass index, alcohol, smoking, vigorous-intensity activity, moderate- intensity activity, night sleep duration, hypertension, diabetes, angina, stroke, chronic lung diseases, and arthritis

Model 4 adjusted the following variables: sex, age, region, urban/rural, married status, alcohol, smoking, vigorous-intensity activity, moderate-intensity activity, body mass index, night sleep duration, hypertension, diabetes, angina, stroke, chronic lung diseases, arthritis, asthma related medications and symptoms (attacks, awakenings and severe shortness of breath)

^#^ *P* < 0.01

### Sarcopenia and comorbidities in asthmatics

In the CHARLS, adjusted OR values in the prevalence of depression respectively were 1.82(95%CI: 1.04–3.18) in nonsevere sarcopenia and 2.02 (95%CI: 1.01–4.10) in severe sarcopenia compared with no sarcopenia after adjusting for demographic characteristics, physical/behavioral factors, and thirteen comorbidities (see Table S3). The presence of sarcopenia was associated with a significantly higher prevalence of depression (adjusted OR = 1.87, 95%CI:1.11–3.14 in model 3).

In the WHO SAGE, sarcopenia was an independent risk factor of COPD in individuals with asthma(adjusted OR = 5.76, 95%CI:2.01–16.5 in model 4). When model 3 added the adjustments of asthmatic medication and symptoms, the prevalence of COPD in the non-severe sarcopenia group (adjusted OR = 9.11, 95%CI:2.69–30.8 in model 4) was significantly higher than that in the no sarcopenia group (see Table S4).

## Discussion

This study used two national population-based surveys to determine the associations between sarcopenia with asthma and found the following results: Firstly, the prevalence of sarcopenia and severe sarcopenia defined by the AWGS 2019 criteria was 12.3–21.3% and 5–5.8% in Chinese older asthmatics. There was no significantly bidirectional association in the prevalence between asthma and sarcopenia, but an upwards trend of sarcopenia was associated with the increasing numbers of asthma-related symptoms. Secondly, the presence of sarcopenia, especially severe sarcopenia, was accompanied by a significantly increased risk of airway obstruction and an obvious reduction of PEF. Thirdly, sarcopenia was positively associated with the prevalence of depression and COPD in terms of asthmatic comorbidity.

Studies on sarcopenia and asthma did not obtain enough attention and are very scant. This was the first study to estimate the prevalence of sarcopenia in Chinese population with asthma by using the AWGS 2019 criteria. Our study demonstrated that approximately 17.6% and 5.5% of asthmatics may be diagnosed with sarcopenia and severe sarcopenia. The prevalence of sarcopenia in the CHARLS was higher than that in WHO SAGE. A possible explanation was that more individuals (62.6%) came from rural in the CHARLS compared with the WHO SAGE (47%). Two analyses based on the CHARLS and WHO SAGE suggested that rural is an independent risk factor of sarcopenia. Previous studies have fully explored the adverse effects of sarcopenia on chronic obstruction pulmonary diseases [21, 22] and health status in the general population [23, 24]. Our study suggested that the prevalence of sarcopenia among asthmatics was higher than that among individuals without asthma. The screening and study of sarcopenia in asthmatics should not be neglected, especially for ageing individuals with rural, depression and airway obstruction.

Persistent airway obstruction and lung function impairment are two important hallmarks of asthmatic deterioration [1]. Airway obstruction and declined lung function not only augment the risk of asthmatic exacerbations and reduce the quality of life, but also increase the difficulty of asthmatic medication treatment. ^1^ Short-term and long-term PEF monitoring may identify expiratory airflow limitations and contribute to early detection of asthmatic exacerbations and the assessment of asthma control levels. In our study, sarcopenia was found to have a positive correlation with the prevalence of airway obstructive and the decline of PEF. We also observed that asthmatics with sarcopenia were more prone to developing COPD than those without sarcopenia. It is reasonable to expect that sarcopenia along with systemic muscle fiber atrophy and weakness will inevitably lead to the functional disability of respiratory muscles. The crosstalk between sarcopenia and adverse health consequences is mediated through complex mechanisms and pathways, including microRNAs, inflammatory processes, oxidative stress and etc. [25–27]. The onset and development of asthma are known to involve the regulation of microRNAs and inflammatory processes [28, 29]. In asthmatics, the expression levels of miR-21 and miR-34 had strongly negative associations with handgrip strength and ASM, whereas miR-133 and miR-206 showed significantly positive correlations with handgrip strength and ASM [30]. Sarcopenia along with low handgrip strength and ASM potentially reduces the levels of miR-133 and miR-206 in asthmatics. The decreasing miR-133a could up-regulate RhoA expression of bronchial smooth muscle in an asthma model, which may lead to an augmentation of bronchial smooth muscle contraction and induce airway hyperresponsiveness (AHR) in individuals with asthma [31]. In a 4,4′-methylene diphenyl diisocyanate induced asthma model, the reduction of miR-206-3p might increase inducible nitric oxide synthase transcription expression by targeting calcineurin/NFAT signaling, in turn leading to AHR. Increasing the level of miR-21 not only promotes the differentiation of T cells towards Th2 in eosinophilic asthma, but also restrains HDAC2 levels and results in glucocorticoids insensitivity by up-regulating PI3K-mediated phosphorylation and nuclear translocation of pAKT in neutrophilic asthma [28]. Multiple studies have shown that sarcopenia is associated with a significant increase in pro-inflammatory cytokines, including TNF-α, IL-6, and C-reactive protein [32]. Taken together, the microRNAs regulatory network and inflammatory processes can be considered the underlying pathophysiology linking sarcopenia with asthma.

Depression is classified as an important independent risk factor of asthma onset, exacerbations and mortality [1]. Our study suggested a bidirectional association between depression and sarcopenia in asthmatics, similar to the results in the general population [33, 34]. Sarcopenia, especially severe sarcopenia, was associated with a significant increase of depression compared with no sarcopenia. Sarcopenia and depression harbor common pathophysiological pathways with regard to inflammatory processes, neurotrophins and oxidative stress [33]. In addition, the onset of sarcopenia and depression may attribute to some same lifestyle behaviors, such as low physical activity, smoking, and malnutrition [33]. Depression caused by sarcopenia is likely to participate in the occurrence and development of asthma.

The main strength of this study was that we assessed the relationships between sarcopenia and asthmatic prevalence, lung function and comorbidities in the Chinese population for the first time by using two national population-based studies. However, several limitations also existed in our study. Firstly, the diagnoses of asthma and some comorbidities depended on the questionnaire's results, which may have resulted in a potential selection bias. Secondly, an anthropometric equation, instead of Dual X-ray absorpometry (DXA) or Bioelectrical impedance analysis(BIA), was used to evaluate ASM. However, this equation has previously been validated in the Chinese population [13]. Our cut-off value of low ASM was substantially identical to that in the study of Wu and the colleagues [16]. Besides, the use of anthropometric equation to assess low ASM may obtain a cost-effective alternative to BIA or DXA for improving the diagnosis of sarcopenia, especially in large-sample population-based studies [35]. Thirdly, the cross-sectional nature of the two studies restrains the capability to infer interactional causation between asthma and sarcopenia.

## Conclusion

This study reports the prevalence of sarcopenia and severe sarcopenia in Chinese older population and asthmatics. Sarcopenia can contribute to the development of asthma by affecting lung function and comorbidity and may be considered a treatable trait of asthma management. When considering the high prevalence and significant effect of sarcopenia, it is worth designing and implementing a routine screening for asthmatics.

## Supplementary Information

**Additional file 1:** **Table S1.** The associations between sarcopenia and asthma in the China Health and Retirement Longitudinal Study. **Table S2.** The associations between sarcopenia and asthma in the Study on global AGEing and adult health from China. **Table S3.** The associations between sarcopenia with PEF and depression among asthmatics in the China Health and Retirement Longitudinal Study. **Table S4.** The associations between sarcopenia and chronic obstructive pulmonary disease among asthmatics in the Study on global AGEing and adult health from China. **S1 Figure.** The screen of independent risk factors of cancer specific death using the least absolute shrinkage and selection operator (LASSO) analysis with binomial regression model in in the China Health and Retirement Longitudinal Study and the Study on global AGEing and adult health from China. A. Tuning parameter selection in the LASSO model used 10-fold cross-validation via minimum criteria in the China Health and Retirement Longitudinal Study; B. LASSO coefficient profiles of potential risk factors in the China Health and Retirement Longitudinal Study; C. The area under the receiver operating characteristic curve (AUC=0.915) in the China Health and Retirement Longitudinal Study; D. Tuning parameter selection in the LASSO model used 10-fold cross-validation via minimum criteria in the Study on global AGEing and adult health from China; E. LASSO coefficient profiles of potential risk factors in the the Study on global AGEing and adult health from China; F. The area under the receiver operating characteristic curve (AUC=0.958) in the Study on global AGEing and adult health from China.

## Data Availability

The data underlying this study were obtained from open CHARLS and SAGE databases. All relevant data are within the paper.

## Abbreviations

AHR: Airway hyperresponsiveness
ASM: Appendicular skeletal muscle mass
AWGS: Asian Working Group for sarcopenia
BIA: Bioelectrical impedance analysis
BMI: Body mass index
CAPI: Computer-assisted personal interview
CHARLS: The China Health and Retirement Longitudinal Study
CI: Confidence interval
DXA: Dual X-ray absorpometry
LASSO: Least absolute shrinkage and selection operator
OR: Odds ratio
RR: Rate ratio
SAGE: Global Ageing and Adult Health
