# Supplementary material for: Associations between sarcopenia with asthmatic prevalence, lung function and comorbidity
Source: BMC Geriatr. 2022 Aug 24;22:703. doi: 10.1186/s12877-022-03394-9 (PMC9404581; doi:10.1186/s12877-022-03394-9)
Supplement: Supplementary file 1 — Additional file 1: Table S1. The associations between sarcopenia and asthma in the China Health and Retirement Longitudinal Study. Table S2. The associations between sarcopenia and asthma in the Study on global AGEing and adult health from China. Table S3. The associations between sarcopenia with PEF and depression among asthmatics in the China Health and Retirement Longitudinal Study. Table S4. The associations between sarcopenia and chronic obstructive pulmonary disease among asthmatics in the Study on global AGEing and adult health from China. S1 Figure. The screen of independent risk factors of cancer specific death using the least absolute shrinkage and selection operator (LASSO) analysis with binomial regression model in in the China Health and Retirement Longitudinal Study and the Study on global AGEing and adult health from China. A. Tuning parameter selection in the LASSO model used 10-fold cross-validation via minimum criteria in the China Health and Retirement Longitudinal Study; B. LASSO coefficient profiles of potential risk factors in the China Health and Retirement Longitudinal Study; C. The area under the receiver operating characteristic curve (AUC=0.915) in the China Health and Retirement Longitudinal Study; D. Tuning parameter selection in the LASSO model used 10-fold cross-validation via minimum criteria in the Study on global AGEing and adult health from China; E. LASSO coefficient profiles of potential risk factors in the the Study on global AGEing and adult health from China; F. The area under the receiver operating characteristic curve (AUC=0.958) in the Study on global AGEing and adult health from China. [file 12877_2022_3394_MOESM1_ESM.docx]

| Table S1: The associations between sarcopenia and asthma in the China Health and Retirement Longitudinal Study | | | | |
| --- | --- | --- | --- | --- |
|  | Model 1 | | Model 2 | Model 3 |
| The associations between sarcopenia with the prevalence of asthma | | | | |
| Sarcopenia ^2^ | |  |  |  |
| No sarcopenia | | Ref | Ref | Ref |
| Sarcopenia | | 1.43(0.98, 1.65) | 1.25(0.99, 1.59) | 1.13(0.87, 1.47) |
| Sarcopenia ^3^ | |  |  |  |
| No sarcopenia | | Ref | Ref | Ref |
| Non-severe sarcopenia | | 1.39(0.89, 2.18) | 1.26(0.98, 1.67) | 1.11(0.84, 1.46) |
| Severe sarcopenia | | 1.48(0.89, 2.46) | 1.23(0.86, 1.74) | 1.20(0.82, 1.74) |
| The association between asthma with the prevalence of sarcopenia | | | | |
| Asthma |  | |  |  |
| No | Ref | | Ref | Ref |
| Yes | 1.25(0.84, 1.89) | | 1.28(1.0, 1.64) | 1.15(0.88, 1.50) |
| Model 1 adjusted the following variables: sex, age, region, urban/rural, married status, and body mass index.  Model 2 adjusted the following variables: sex, age, region, urban/rural, married status, body mass index, alcohol, smoking and night sleep duration.  Model 2 adjusted the following variables: sex, age, region, urban/rural, married status, alcohol, smoking, body mass index, night sleep duration, hypertension, dyslipidemia, hyperglycemia, cancers, chronic lung diseases, liver diseases, heart diseases, stroke, kidney diseases , digestive diseases, emotional or psychiatric problems, memory-related diseases, arthritis or rheumatism, and depression. | | | | |

| Table S2: The associations between sarcopenia and asthma in the Study on global AGEing and adult health from China | | | |
| --- | --- | --- | --- |
|  | Model 1 | Model 2 | Model 3 |
| The associations between sarcopenia with the prevalence of asthma | | | |
| Sarcopenia ^2^ |  |  |  |
| No sarcopenia | Ref | Ref | Ref |
| Sarcopenia | 1.43(0.98, 2.09) | 1.38(0.88, 2.17) | 1.29(0.87, 1.93) |
| Sarcopenia ^3^ |  |  |  |
| No sarcopenia | Ref | Ref | Ref |
| Non-severe sarcopenia | 1.39(0.89, 2.18) | 1.38(0.88, 2.17) | 1.32 (0.82, 2.11) |
| Severe sarcopenia | 1.48(0.89, 2.46) | 1.45(0.87, 2.42) | 1.26(0.74, 2.15) |
| The association between asthma with the prevalence of sarcopenia | | | |
| Asthma |  |  |  |
| No | Ref | Ref | Ref |
| Yes | 1.26(0.84, 1.87) | 1.27(0.85, 1.90) | 1.14(0.75, 1.72) |
| Model 1 adjusted the following variables: sex, age, region, urban/rural, married status and body mass index.  Model 2 adjusted the following variables: sex, age, region, urban/rural, married status, body mass index, alcohol, smoking, vigorous-intensity activity , moderate- intensity activity and night sleep duration.  Model 3 adjusted the following variables: sex, age, region, urban/rural, married status, body mass index, alcohol, smoking, vigorous-intensity activity , moderate- intensity activity, night sleep duration, hypertension, diabetes, angina, stroke, chronic lung diseases, and arthritis. | | | |

| Table S3: The associations between sarcopenia with PEF and depression among asthmatics in the China Health and Retirement Longitudinal Study | | | |
| --- | --- | --- | --- |
|  | Model 1 | Model 2 | Model 3 |
| The associations between sarcopenia with PEF | | | |
| Sarcopenia ^2^ |  |  |  |
| No sarcopenia | Ref | Ref | Ref |
| Sarcopenia | 0.85(0.83, 0.86) | 0.86(0.85, 0.88) | 0.88(0.87, 0.90) |
| Sarcopenia ^3^ |  |  |  |
| No sarcopenia | Ref | Ref | Ref |
| Non-severe sarcopenia | 0.90(0.88, 0.91) | 0.92(0.90, 0.94) | 0.926(0.91, 0.934) |
| Severe sarcopenia | 0.72(0.70, 0.74) | 0.72(0.70, 0.74) | 0.76(0.74, 0.78) |
| The association between asthma with the prevalence of depression | | | |
| Sarcopenia ^2^ |  |  |  |
| No sarcopenia | Ref | Ref | Ref |
| Sarcopenia | 1.77(1.09, 2.89) | 1.84(1.10, 3.06) | 1.87(1.11, 3.14) |
| Sarcopenia ^3^ |  |  |  |
| No sarcopenia | Ref | Ref | Ref |
| Non-severe sarcopenia | 1.76(1.03, 2.98) | 1.81(1.04, 3.13) | 1.82(1.04, 3.18) |
| Severe sarcopenia | 1.80(0.91, 3.59)^$^ | 1.91(0.93, 3.95)^$^ | 2.02(1.01, 4.10) |
| Model 1 adjusted the following variables: sex, age, region, urban/rural, married status, and body mass index.  Model 2 adjusted the following variables: sex, age, region, urban/rural, married status, body mass index, alcohol, smoking and night sleep duration.  Model 2 adjusted the following variables: sex, age, region, urban/rural, married status, alcohol, smoking, body mass index, night sleep duration, hypertension, dyslipidemia, hyperglycemia, cancers, chronic lung diseases, liver diseases, heart diseases, stroke, kidney diseases , digestive diseases, emotional or psychiatric problems, memory-related diseases, arthritis or rheumatism, and depression.  PEF, peak expiratory flow;  ^$^, *P* > 0.05 | | | |

| Table S4: The associations between sarcopenia and chronic obstructive pulmonary disease among asthmatics in the Study on global AGEing and adult health from China | | | | |
| --- | --- | --- | --- | --- |
|  | Model 1 | Model 2 | Model 3 | Model 4 |
| Sarcopenia ^2^ |  |  |  |  |
| No sarcopenia | Ref | Ref | Ref | Ref |
| Sarcopenia | 5.39(2.14, 13.6) | 5.41(2.03, 14.5) | 5.18(1.90, 14.1) | 5.76(2.01, 16.5) |
| Sarcopenia ^3^ |  |  |  |  |
| No sarcopenia | Ref | Ref | Ref | Ref |
| Non-severe sarcopenia | 7.37(2.62, 20.7) | 8.01(2.63, 24.4) | 7.38(2.37, 22.9) | 9.11(2.09, 30.8) |
| Severe sarcopenia | 3.07(0.90, 10.5)^$^ | 2.79(0.76, 10.2)^$^ | 2.84(0.76, 10.7)^$^ | 2.92(0.75, 11.4)^$^ |
| Model 1 adjusted the following variables: sex, age, region, urban/rural, married status and body mass index.  Model 2 adjusted the following variables: sex, age, region, urban/rural, married status, body mass index, alcohol, smoking, vigorous-intensity activity , moderate- intensity activity and night sleep duration.  Model 3 adjusted the following variables: sex, age, region, urban/rural, married status, body mass index, alcohol, smoking, vigorous-intensity activity , moderate- intensity activity, night sleep duration, hypertension, diabetes, angina, stroke, chronic lung diseases, and arthritis.  Model 4 adjusted the following variables: sex, age, region, urban/rural, married status, alcohol, smoking, vigorous-intensity activity , moderate-intensity activity, body mass index, night sleep duration, hypertension, diabetes, angina, stroke, chronic lung diseases, arthritis, asthma related medications and symptoms (attacks, awakenings and severe shortness of breath).  ^$^ P >0.0.5 | | | | |


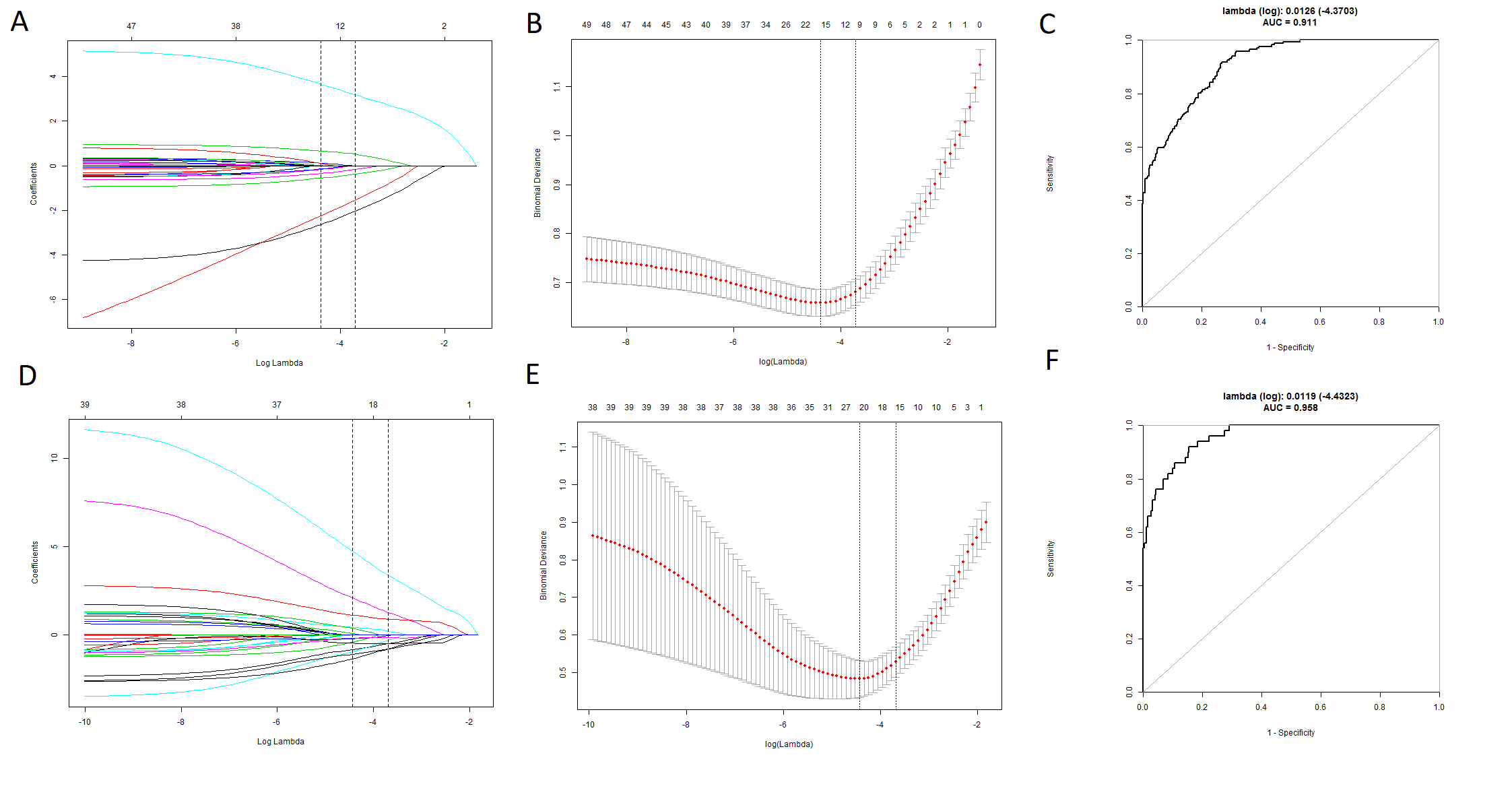


S1 Figure. The screen of independent risk factors of cancer specific death using the least absolute shrinkage and selection operator (LASSO) analysis with binomial regression model in in the China Health and Retirement Longitudinal Study and the Study on global AGEing and adult health from China. A. Tuning parameter selection in the LASSO model used 10-fold cross-validation via minimum criteria in the China Health and Retirement Longitudinal Study; B. LASSO coefficient profiles of potential risk factors in the China Health and Retirement Longitudinal Study; C. The area under the receiver operating characteristic curve (AUC=0.915) in the China Health and Retirement Longitudinal Study; D. Tuning parameter selection in the LASSO model used 10-fold cross-validation via minimum criteria in the Study on global AGEing and adult health from China; E. LASSO coefficient profiles of potential risk factors in the the Study on global AGEing and adult health from China; F. The area under the receiver operating characteristic curve (AUC=0.958) in the Study on global AGEing and adult health from China.
